# mTORC1 regulates apoptosis and cell proliferation in pterygium via targeting autophagy and FGFR3

**DOI:** 10.1038/s41598-017-07844-y

**Published:** 2017-08-04

**Authors:** Yanli Liu, Hanchun Xu, Meixia An

**Affiliations:** Department of Ophthalmology, The Third Affiliated Hospital, Southern Medical University, Guangzhou, China

## Abstract

Pterygium is one of the most common ocular surface diseases. During the initiation of pterygium, resting epithelial cells are activated and exhibit aberrant apoptosis and cell proliferation. Mechanistic target of rapamycin complex 1 (mTORC1) is a central regulator of cell growth, cell proliferation, protein synthesis, autophagy and transcription. However, the effect of mTORC1 activation in epithelial cells on pterygium development has not yet been reported. Additionally, the roles of mTORC1 in aberrant apoptosis and cell proliferation during the initiation of pterygium, and the underlying mechanisms, are not known. Herein, we evaluated mTOR signalling in pterygium growth and development. The results revealed that mTOR signalling, especially mTORC1 signaling, is highly activated, and aberrant apoptosis and cell proliferation were observed in pterygium. mTORC1 activation inhibits apoptosis in pterygium by regulating Beclin 1-dependent autophagy via targeting Bcl-2. mTORC1 also negatively regulates fibroblast growth factor receptor 3 (FGFR3) through inhibition of p73, thereby stimulating cell proliferation in pterygium. These data demonstrate that mTORC1 signalling is highly activated in pterygium and provide new insights into the pathogenesis and progression of pterygium. Hence, mTORC1 may be a novel therapeutic target for the treatment of pterygium.

## Introduction

Pterygium is a common ocular surface disease that leads to corneal astigmatism, dyskinesia and even vision loss. Chronic ultraviolet (UV) exposure is the main risk of pterygium, and it promotes the overgrowth of abnormal conjunctiva on the cornea^1^. Pterygium is characterised by epithelial cell hyperplasia with an underlying stroma of fibrovascular proliferation, inflammatory infiltrates, neovascularisation, and extracellular matrix remodelling^2^. Although numerous studies have been performed to further characterise pterygium pathogenesis, the exact mechanisms of pterygium growth and development remain unkown.

Recent studies have shown that pterygium epithelial cells and the fibrovascular layer express the anti-apoptosis protein Bcl-2 as well as molecules associated with proliferation, exhibiting elevated CyclinD1 and decreased p27 (KIP1), thus indicating that both apoptosis and cell proliferation are predominantly involved in the progression of pterygium^3, 4^. However, more considerable progress must be made towards understanding the mechanisms responsible for regulating pterygium epithelial apoptosis and cell proliferation.

Mammalian target of rapamycin (mTOR) is an important suppressor of autophagy and regulator of cell metabolism. mTOR forms two distinct multiprotein complexes: mammalian target of rapamycin complex 1 (mTORC1) and mTORC2. mTOR interacts with Raptor, mLst8/GbL, Deptor, and PRAS40, thereby forming mTOR complex 1 (mTORC1). mTORC1 is a sensitive target of rapamycin and integrates input from many upstream signals, including insulin, growth factors, amino acids, oxygen, and energy levels, mTORC1 is a central regulator of cell growth, cell proliferation, cell motility, cell survival, protein synthesis, autophagy and transcription^5^. In skin cancer, unbalanced mTOR signalling leads to UV-induced hyperproliferation and malignant transformation, thus indicating that mTOR may be involved in pterygium pathology^6^. Other studies have found that rapamycin inhibits corneal neovascularisation^7^, transplant rejection^8^ and the epithelial-to-mesenchymal transition (EMT) of the lens epithelium^9^. Moreover, rapamycin has also been reported to promote the apoptosis of human lens epithelial cells (LECs) and to inhibit the proliferation of rabbit LECs^10–12^. Therefore, we speculate that mTOR may play an essential role in pterygium pathology and may represent a potential treatment for pterygium.

In this study, we estimated mTOR signalling and demonstrated the roles of autophagy and fibroblast growth factor receptor 3 (FGFR3) in aberrant apoptosis and hyperproliferation in pterygium. Our findings establish a novel link between mTOR, autophagy, FGFR3 and the aberrant apoptosis and hyperproliferation that occurs during the growth and development of pterygium.

## Results

### mTORC1 is highly activated in pterygium compared with normal conjunctiva

**Figure 1:**
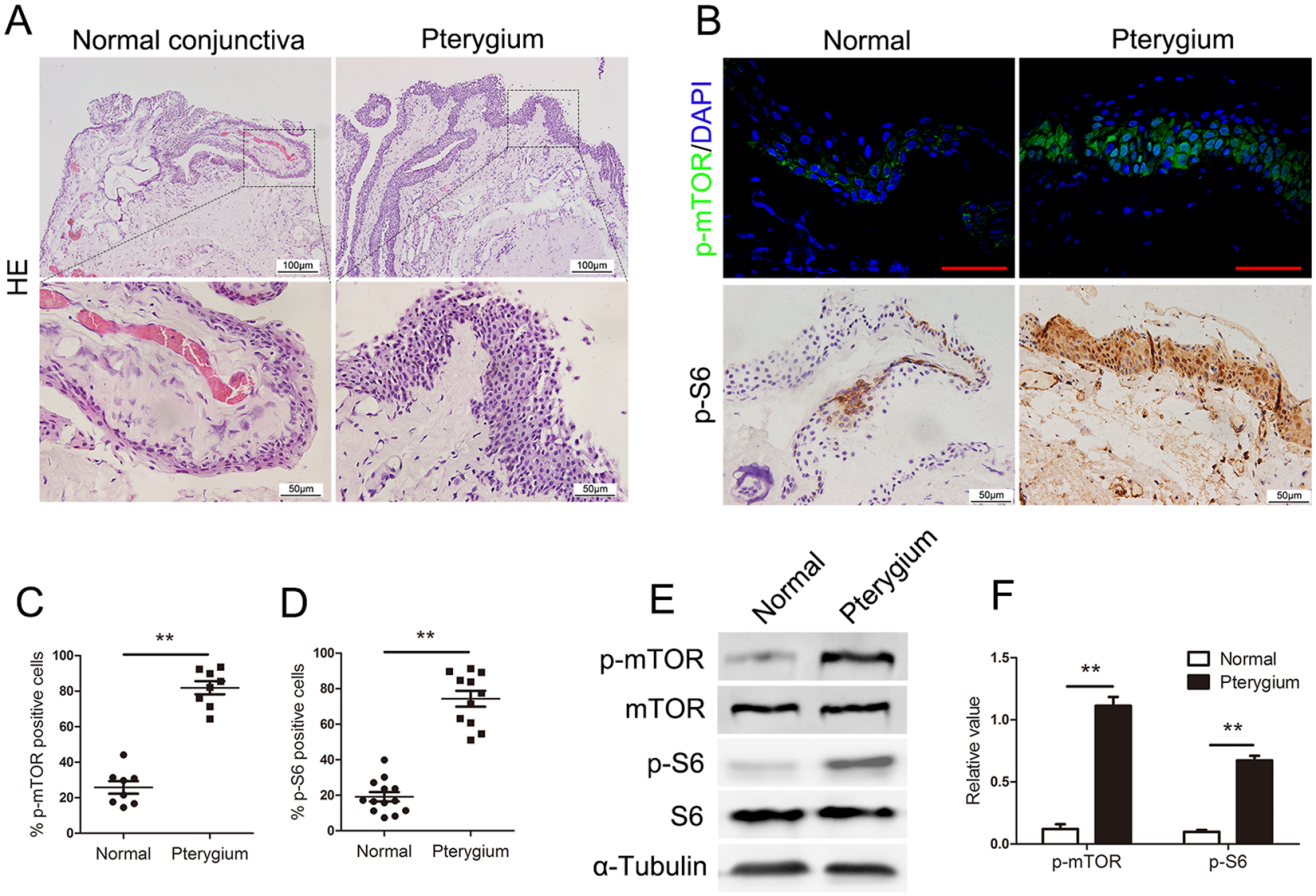
mTORC1 is activated in pterygium epithelial cells. (**A**) H&E staining in pterygium and normal conjunctiva, Scale bar: 50 μm. Higher magnification is shown on the bottom, Scale bar: 100 μm. (**B**) p-mTOR (Ser2448) IF staining (b-top) and p-S6 (S235/236) IHC staining (b-bottom) in pterygium and normal conjunctiva, Scale bar: 50 μm. p-mTOR positive cells (**C**) and p-S6 positive cells (**D**) were significantly increased in pterygium compared with normal conjunctiva. mTOR and mTORC1 activation were confirmed by western blotting (e and f). **p < 0.01.

Human primary pterygium and normal conjunctiva were isolated to investigate the morphological characteristics after removal surgery. Histological staining showed well-defined basement membrane between the epithelium and the underlying stroma in normal conjunctiva. The pterygium tissue exhibited epithelial cell hyperplasia with more than 10 layers followed by a trailing stroma of enriched fibrovascular, inflammatory cells and blood vessels, whereas these findings were observed to a markedly lesser extent in normal conjunctiva (Fig. 1A).

We next determined whether mTOR was activated in pterygium compared with normal conjunctiva. Immunofluorescence analysis revealed significantly upregulated p-mTOR protein expression in human pterygium compared with normal conjunctiva (Fig. 1B,C). Intriguingly, enhanced phosphorylation of S6 (S235/236) (a downstream effector of mTORC1 and S6K1) was observed in all epithelial cell layers of pterygium, but was detected in only the basal layer of epithelium cells in normal conjunctiva (Fig. 1B,D). Furthermore, these results were confirmed by western blotting analysis (Fig. 1E,F). These observations demonstrated that mTOR signalling, and mTORC1 in particular, is highly activated in pterygium.

### Aberrant apoptosis and cell proliferation occurs in pterygium

**Figure 2:**
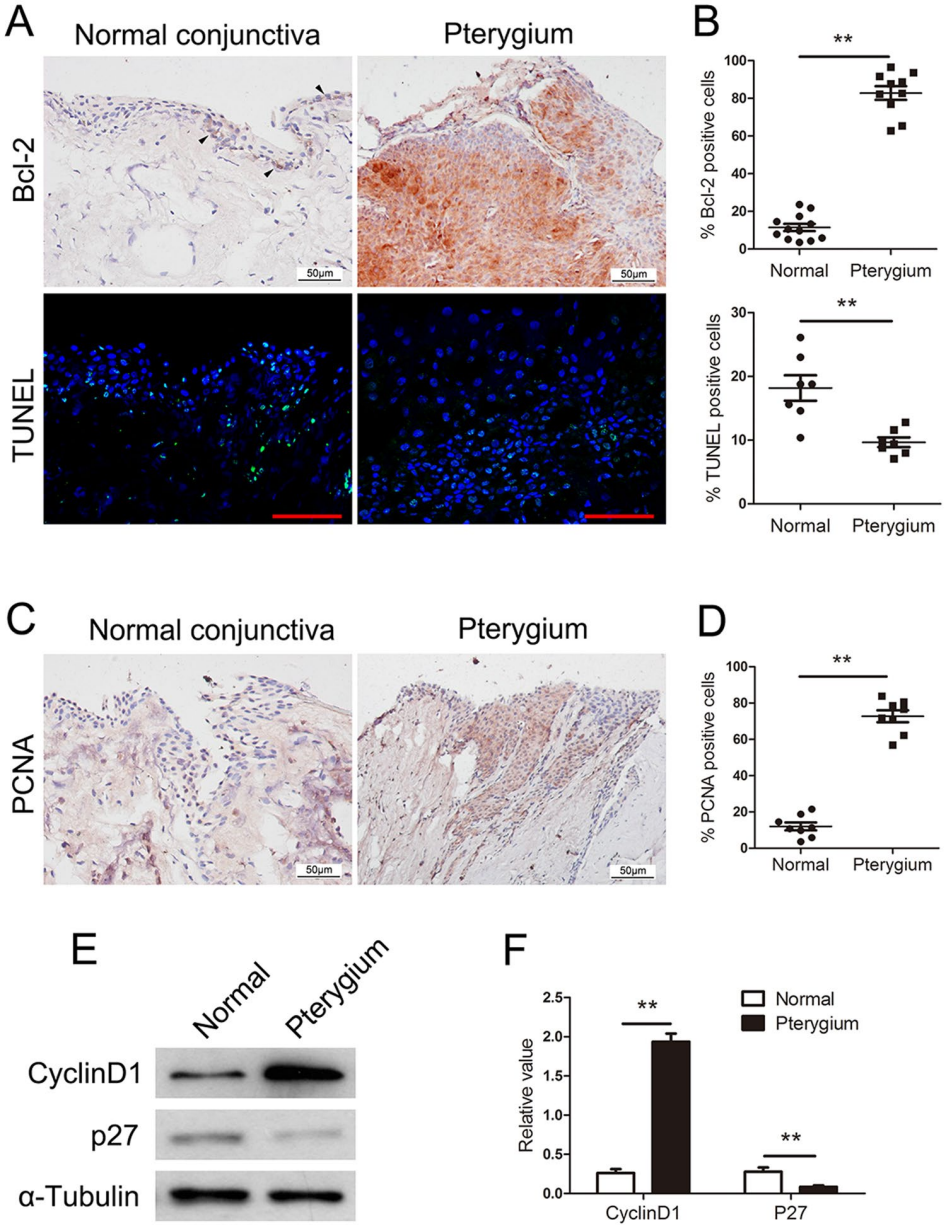
Aberrant apoptosis and cell proliferation occurs in pterygium. (**A** and **B**) Immunohistochemical and immunofluorescence analysis of Bcl-2 and TUNEL analysis revealed a significant decrease in the percentage (%) of apoptotic cells in human pterygium compared with normal conjunctiva. Immunohistochemical analysis of PCNA (**C** and **D**) and western blotting analysis of CyclinD1 and P27 (**E** and **F**) showed hyperproliferation in perygium compared with normal conjunctiva. Triangles signify indicate the most positive cells. Scale bar: 50 μm. **p < 0.01.

To investigate the underlying cellular mechanisms, we examined apoptosis and cell proliferation in epithelial cells, because these processes are essential for pterygium growth and development which are blocked in normal conjunctiva. Measurement of Bcl-2 expression and transferase-mediated dUTP nick-end labelling (TUNEL) analysis were used to detect apoptotic cells. Bcl-2 is involved in the response to apoptosis and considered an antiapoptotic protein. Immunochemical staining showed that Bcl-2-positive cells were nearly undetectable in normal conjunctiva, but were significantly increased and expressed across the entire width of the epithelial layer in pterygium when the epithelial hyperplasia was florid. Using immunofluorescence staining, we observed TUNEL-positive cells throughout the thickness of the epithelial layer in normal conjunctiva. However, there were few TUNEL-positive cells detected in pterygium (Fig. 2A,B).

We further investigated the expression of the proliferation related genes PCNA, CyclinD1 and P27 in pterygium and normal conjunctiva. In agreement with findings from other studies, PCNA-positive cells were significantly increased in pterygium (Fig. 2C,D) and western blot analysis of CyclinD1 and P27 confirmed the hyperproliferation characteristic of pterygium compared with normal conjunctiva (Fig. 2E,F). These observations demonstrated that apoptosis and cell proliferation were involved in the pathogenesis of pterygium.

### Autophagy was inhibited by mTORC1 in pterygium and regulated epithelial cell apoptosis

**Figure 3:**
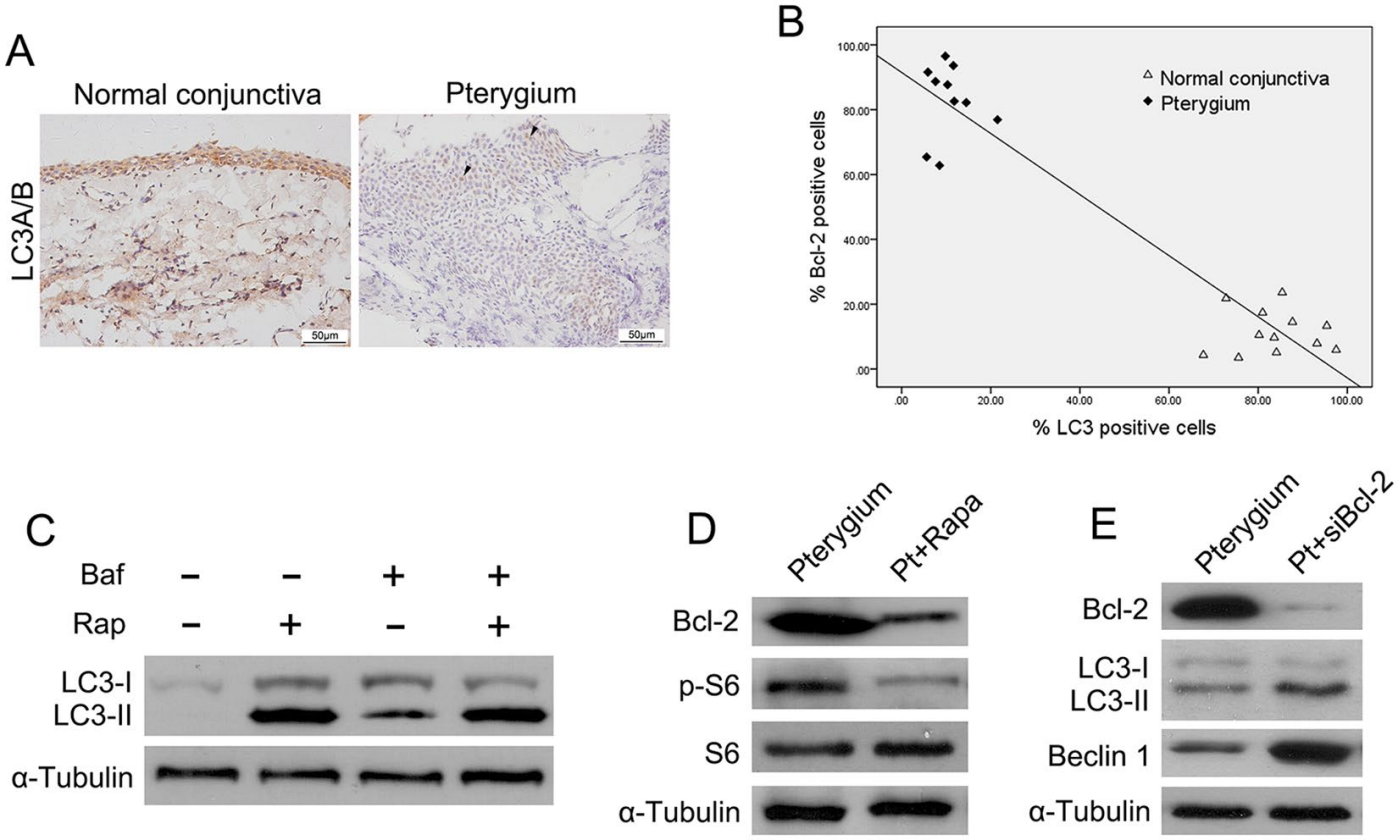
Activation of mTORC1 in pterygium regulates apoptosis by inhibiting Beclin 1 - dependent autophagy. (**A**) LC3-positve cells were observed in all epithelial cell layers of normal conjunctiva, whereas scarely any LC3-positive cells were detected in pterygium. (**B**) A statistically significant inverse correlation between LC3 and Bcl-2-positve cells (Pearson’s correlation: r = −0.953, P < 0.01) was observed. (**C**) Western blot analysis of LC3 in cultured pterygium epithelial cells after treatment of rapamycin (100 nM), bafilomycin A1 (50 nM) or both. (**D**) Western blot analysis of Bcl-2 and p-S6 in cultured pterygium epithelial cells after rapamycin treatment. (**E**) Western blot analysis of Bcl-2, LC3 and Beclin 1 in cultured pterygium epithelial cells transfected with *Bcl-2* siRNA. Scale bar, 50 μm.

Autophagy is essential for cell survival, differentiation, development and homeostasis^13^. Recent studies have highlighted the autophagy-mediated apoptosis in the epithelium^14^, and mTOR inhibition plays protective role in epithelial cell survival by enhancing autophagy^15^. In this study, we detected the direct role of mTORC1 and autophagy in regulating apoptosis of pterygium. Immunohistochemistry showed that LC3 (an autophagy structural and functional factor)-positive cells were significantly decreased in pterygium compared with normal conjunctiva, thus indicating strong inhibition of autophagy in pterygium (Fig. 3A). Further investigation using Pearson’s correlation analysis revealed a statistically significant inverse correlation between LC3 and Bcl-2 expression (Fig. 3B, r = −0.953, P < 0.01). For further assessment of autophagy, we detected the LC3 I-II levels in cultured pterygial cells exposed to bafilomycin A1, rapamycin (an mTORC1-specific inhibitor) or both. Treatment of pterygial cells with both rapamycin and bafilomycin A1 increased conversion of LC3 II from LC3 I, similarly to rapamycin treatment alone, thus indicating enhanced autophagic flux by rapamycin (Fig. 3C). Interestingly, rapamycin treatment not only rescued LC3 expression in pterygium epithelial cells but also downregulated Bcl-2 expression (Fig. 3D).

We next examined the exact relationship between autophagy and apoptosis by transfecting pterygium epithelial cells with Bcl-2 siRNA (50 nmol/l, 48 hours). Western blot analysis revealed that Bcl-2 siRNA significantly inhibited Bcl-2 expression in pterygium epithelial cells. Furthermore, Bcl-2 silencing significantly increased LC3II expression and also upregulated Beclin-1 expression (an autophagy promoting protein) (Fig. 3D). These results indicated that mTORC1 activation regulates apoptosis of pterygium by inhibiting Beclin 1 -- dependent autophagy.

### Downregulation of FGFR3 expression in pterygium epithelial cells

**Figure 4:**
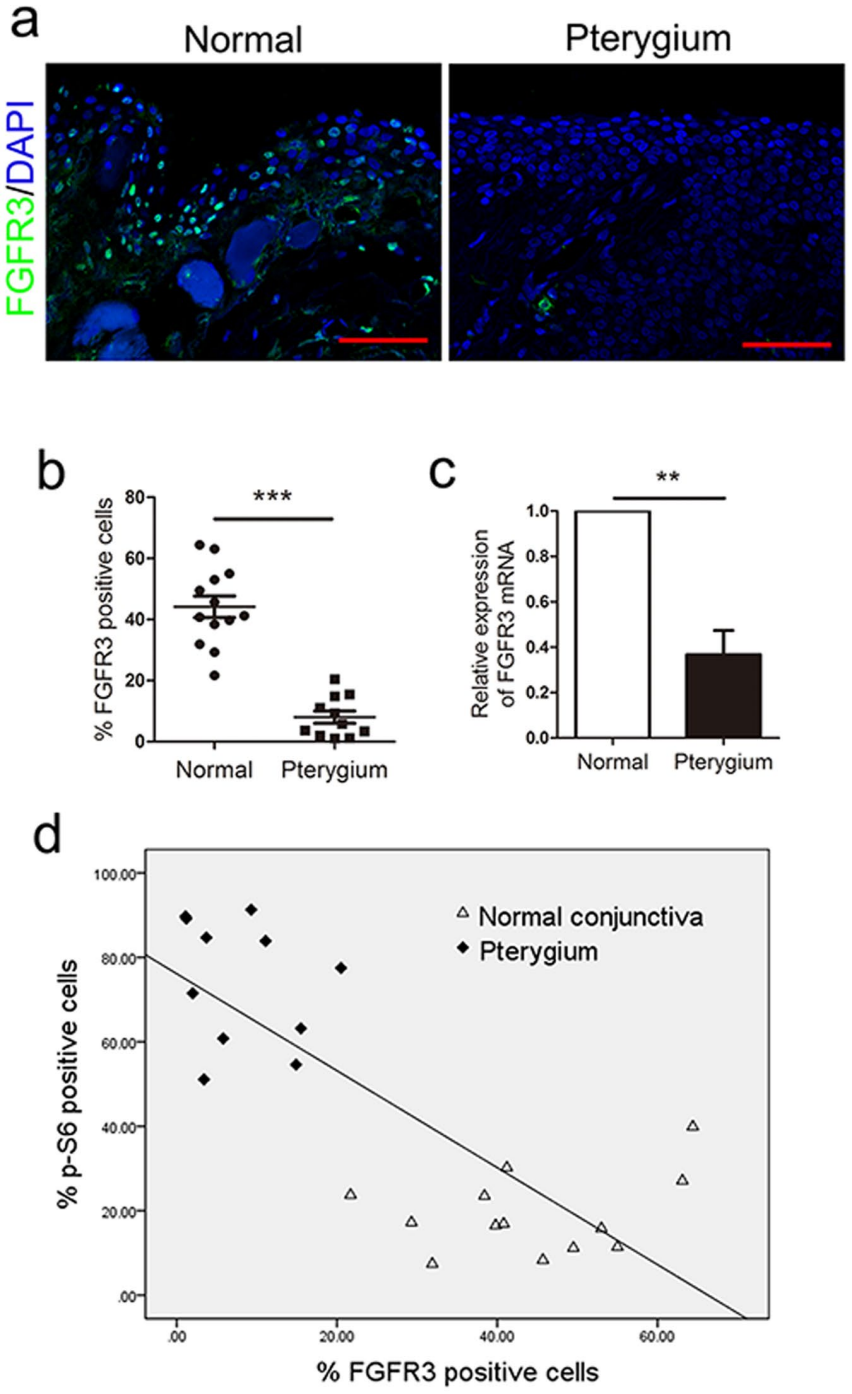
FGFR3 expression is decreased in pterygium. (**a** and **b**) Immunofluorescence staining and analysis of FGFR3 in pterygium compared with normal conjunctiva. (**c**) qPCR analysis of FGFR3 mRNA expression in pterygium and normal conjunctiva. (**d**) A statistically significant inverse correlation between p-S6 and FGFR3-positve cells (Pearson’s correlation: r = −0.791, P < 0.01) was observed. Scale bar: 50 μm. **p < 0.01, ***p < 0.001.

We further explored the molecular mechanisms that stimulate pterygium cell proliferation. Among the multiple signalling pathways regulating cell development, FGFR3 plays a central role in epithelial cell proliferation via mediating FGF signalling^16^. On the basis of immunofluorescence analysis, FGFR3 was found to be expressed across the entire width of the epithelial layer in normal conjunctiva, whereas FGFR3 was scarcely detected in pterygium (Fig. 4a,b). Moreover, FGFR3 mRNA levels were also markedly decreased in pterygium compared with normal conjunctiva (Fig. 4c). Pearson’s correlation analysis revealed a strong association between FGFR3 and p-S6 (S235/236) expression levels (Fig. 4d, r = −0.791, P < 0.01). These findings suggested that FGFR3 expression was downregulated in pterygium and was inversely correlated with mTORC1 activation.

### mTORC1 activation inhibited FGFR3, thus promoting cell proliferation in pterygium by targeting p73

**Figure 5:**
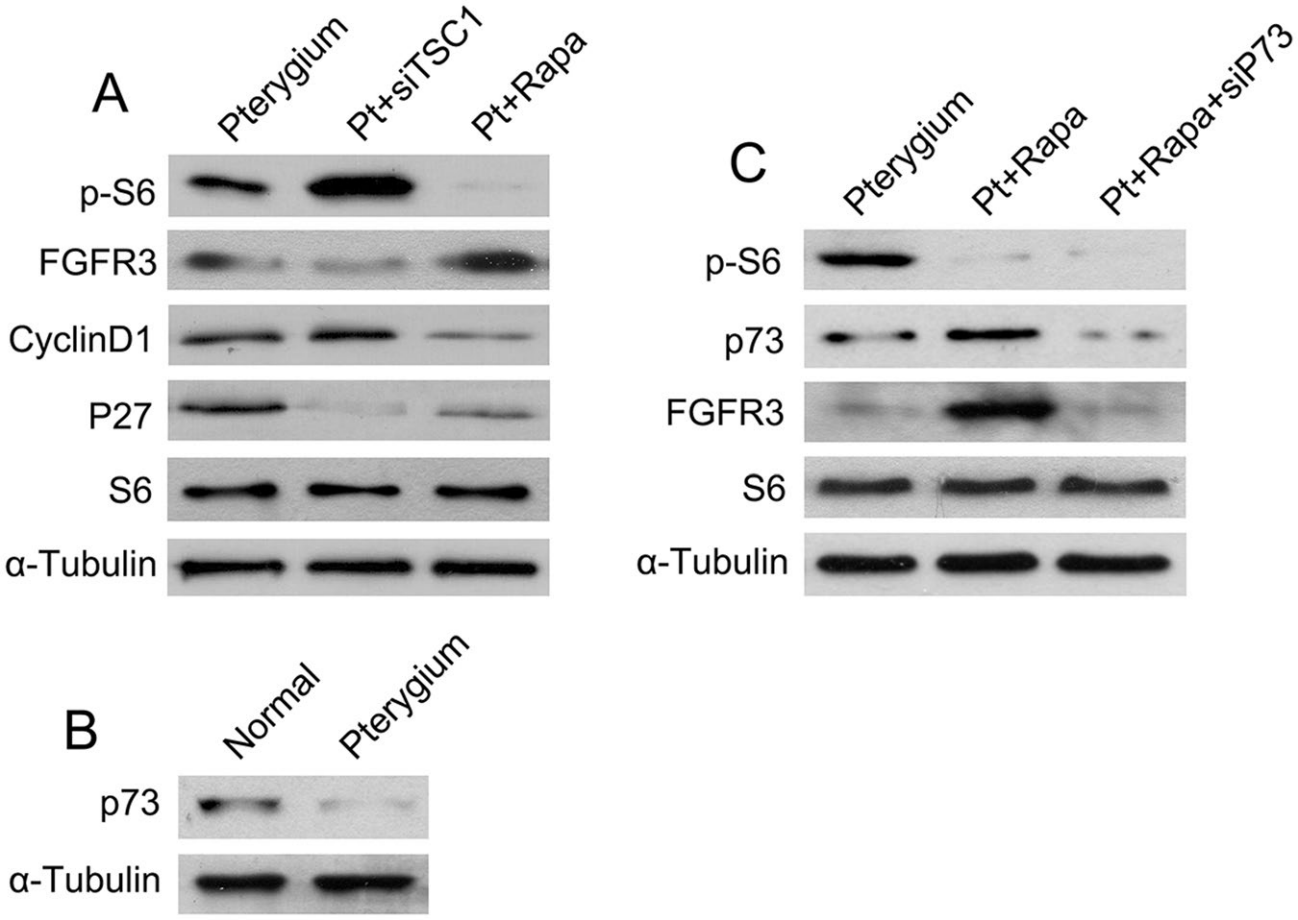
mTORC1 activation in pterygium inhibited FGFR3, thus promoting cell proliferation by targeting p73. (**A**) Western blot analysis in cultured pterygium epithelial cells after treatment of *TSC1* siRNA or rapamycin. (**B**) p73 expression in pterygium and normal conjunctiva. (**C**) Western blot analysis of p-S6, p73 and FGFR3 in cultured pterygium epithelial cells after treatment of rapamycin with or without *p73* siRNA transfection.

Investigation into the molecular mechanisms of mTORC1 and FGFR3 revealed that rapamycin administration rescued the FGFR3 expression and the negative effect of FGFR3 on proliferation of pterygium epithelial cells (Fig. 5A). Furthermore. we found that p73, a transcription factor for FGFR3 that is regulated by mTORC1^17, 18^, was also downregulated in pterygium compared with normal conjunctiva, on the basis of western blot analysis (Fig. 5B). Rapamycin enhanced p73 and FGFR3 expression levels in pterygium epithelial cells, whereas p73 siRNA inhibited rapamycin-induced FGFR3 expression (Fig. 5C). Together, these results indicated that mTORC1 activation in pterygium epithelial cells negatively regulates FGFR3 through inhibition of p73.

## Discussion

**Figure 6:**
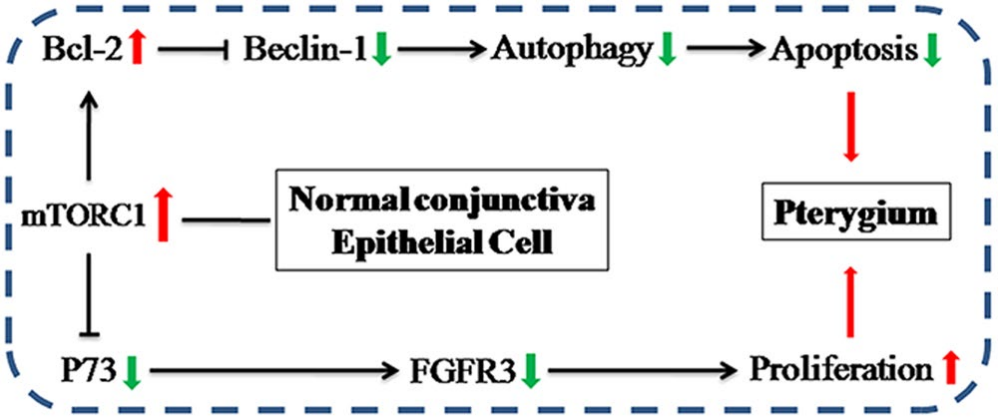
A schematic model depicting the role of mTORC1 in the regulation of pterygium apoptosis and cell proliferation via targeting autophagy and FGFR3.

This study demonstrated the role of mTORC1 signalling in pterygium growth and development. We propose a pathway in which mTORC1 activation downregulates autophagy and FGFR3 in pterygium epithelial cells, thus consequently inhibiting apoptosis, stimulating cell proliferation, and promoting pterygium growth and development. mTORC1 signalling - control of autophagy and FGFR3 is a critical regulator of pterygium epithelial cell metabolism and a potential target for pterygium treatment (Fig. 6).

mTOR is an intracellular coordinator of cell growth and metabolism and has been shown to be associated with various ocular diseases, ranging from corneal to uveitic to retinal. studies have shown that mTOR signalling plays an important role in UV-induced malignant transformation^6^, EMT of the lens epithelium^9^, and apoptosis and cell proliferation of LECs^10–12^, which are also involved in pterygium pathology. To our knowledge, the role of mTOR signalling in pterygium and its potential mechanism is still unknown. We found that mTOR is overexpressed in human pterygium (compared with normal conjunctiva) and that, mTORC1 is activated in particular, with highly enhanced p-S6 (S235/236) expression. Further investigation revealed that mTORC1 activation is significantly associated with inhibition of autophagy and FGFR3 in pterygium.

Autophagy is an essential homeostatic process that delivers cytoplasmic proteins to lysosomes for degradation, and this process is negatively regulated by mTORC1^19^. Dysregulation of autophagy, which results in apoptosis and necrosis, is involved in the occurrence of ocular diseases, including DR, ocular cancers, and glaucoma. In the present study, we found that autophagy is strongly inhibited in pterygium compared with normal conjunctiva and that autophagy is significantly inversely correlated with the expression of Bcl-2, an anti-apoptotic member of the Bcl-2 family that is involved in cell survival^20^. We hypothesized that the inhibition of autophagy in pterygium might be associated with abnormal apoptosis. Interestingly, rapamycin rescued the inhibition of autophagy and the increased expression of Bcl-2 in cultured pterygium epithelial cells. A previous study has shown that Bcl-2 also inhibits the autophagic process via its inhibitory interaction with Beclin 1, an autophagy-promoting protein^21^. Activated autophagy with increased Beclin 1 expression was observed when Bcl-2 was silenced by siRNA in cultured pterygium epithelial cells. Our results demonstrated that the negative effect of activated mTORC1 in the apoptosis of pterygium epithelial cells occurs, at least in part, through the inhibition of Beclin 1-dependent autophagy by targeting Bcl-2.

In this study, we also invesgated the expression of the proliferation related genes PCNA, CyclinD1 and P27 in pterygium and normal conjunctiva. The results indicated hyperproliferation of pterygium epithelial cells. Interestingly, we observed significant inhibition of FGFR3 in pterygium. In normal conjunctiva, FGFR3 was expressed across the entire width of the epithelial layer, whereas in pterygium, FGFR3 was scarcely detected. FGFR3 is a receptor tyrosine kinase of FGF (FGF2 and FGF18) signalling and is generally considered an inhibitor of normal tissue^22^. The critical role of FGFR3 in bone and cartilage development has been elegantly described^23^. In recent years, FGFR3-mediated signalling has also been implicated in other tissues. A previous study has shown that FGFR3 has a negative effect on pancreatic epithelial cell proliferation^16^. In human epithelial ovarian cancer (EOC) cells, miR-99a suppresses EOC cell proliferation by targeting FGFR3^24^. A study on human diabetic corneas has revealed that decreased FGFR3 in the corneal epithelium contributes to impaired corneal cell motility^25^. However, no studies have focused on the effect of FGFR3 in pterygium epithelial cells. In this study, we found that mTORC1 activation decreased FGFR3 expression whereas rapamycin increased FGFR3 expression in pterygium epithelial cells. mTORC1 downregulated FGFR3 transcription through inhibition of p73^18^. On the basis of these data, we propose that loss of FGFR3 in pterygium epithelial cells may result from mTORC1 activation, thus representing a mechanism for aberrant proliferation during pterygium growth and development.

In summary, our study revealed a novel pathway explaining the crucial role of mTORC1 in the pathogenesis of pterygium. mTORC1 activation promotes pterygium growth and development via targeting autophagy and FGFR3, thus inhibiting apoptosis and stimulating cell proliferation. These findings indicate that mTORC1 might be a novel therapeutic target for the treatment of pterygium. However, we have not detected mTORC2 signalling in pterygium, and the exact roles of mTOR signalling in inflammatory infiltrates, neovascularisation and extracellular matrix remodelling during pterygium development remain unclear. Additional studies need to be performed in the future.

## Methods

### Ethics statements

This study was approved by the Ethics Committee of the Third Affiliated Hospital of Southern Medical University. All patients gave informed consent to use their clinical information for scientific research. The procedures and all methods were performed in accordance with the relevant guidelines and regulations of the Ethics Committee of the Third Affiliated Hospital of Southern Medical University.

### Patients and tissues

Pterygium tissues were harvested from 16 patients with primary pterygium undergoing removal surgery (aged 60.88 ± 2.46 years, 7 males and 9 females). Normal conjunctival tissues were collected from 13 patients who underwent cataract surgery (aged 67.92 ± 1.73 years, 7 males and 6 females). All samples were obtained from the Third Affiliated Hospital of Southern Medical University, Guangzhou, China.

### Antibodies

The following antibodies were used: rabbit anti-pS6 (S235/236) (IHC, WB, CST, 4858, USA), rabbit anti-Bcl-2 (IHC, WB, Proteintech, 12789-1-AP, USA), rabbit anti-PCNA (IHC, Bioworld, BS5842, USA), rabbit anti-LC3 (IHC, WB, CST, D3U4C, 12741, USA), rabbit anti-p-mTOR (Ser2448) (IF, WB, CST, 2971, USA), and rabbit anti-FGFR3 (IF, WB, Bioworld, BS1125, USA). For western blotting, antibodies to the following proteins were used: CyclinD1 (WB, CST, 2978, USA), P27 (WB, CST, 2402, USA), Beclin 1 (WB, CST, 3738, USA), and P73 (WB, ABclonal, A2871, USA).

### TUNEL analysis

Pterygium and normal conjunctival tissues were isolated, and the tissues were fixed using 4% paraformaldehyde in PBS at 4 °C for 24 hours. The tissues were embedded in paraffin, and three-micrometre sections were prepared for histological analyses^3, 26^. Apoptosis of pterygium epithelial cells was evaluated by terminal deoxynucleotidyl TUNEL assays using a commercial kit (Promega) according to the manufacturer’s instructions. The sections were viewed and analysed using FluoView FV1000 fluorescence microscope (Olympus, Japan). Three visual fields per sample were randomly selected and observed under 400× magnification.

### Quantitative reverse transcription-polymerase chain reaction

Total RNA was extracted from pterygium and normal conjunctival tissues with TRIzol reagent (Invitrogen). To analyse *FGFR3* (forward primer 5′-GAT GGA CAA GAA GCT GCT GG-3′ and reverse primer 5′-TGC CAA ACT TGT TCT CCA CG-3′), *GAPDH* (forward primer 5′-CTG TTC GAC AGT CAG CCG CAT C-3′ and reverse primer 5′-GCG CCC AAT ACG ACC AAA TCC G-3′) levels, we used TaKaRa reverse transcription reagents and Real-Time PCR Mix (TaKaRa) on a Light Cycler (Roche) intrument. *GAPDH* was used as an endogenous control to normalise for differences in the amount of total RNA.

### Cell isolation and culture

The primary pterygium epithelial cells were isolated as previously described^4^. Briefly, fresh pterygial specimens were cut into small pieces (1–2 mm in diameter) under a stereomicroscope, cultured in Dulbecco’s modified Eagle’s medium (DMEM, Gibco, USA) supplemented with 10% foetal bovine serum and 1% penicillin-streptomycin and cultured in a 5% CO_2_ incubator at 37 °C. When a sufficient number of epithelial cells had surrounded each explant (usually after 7–10 days), we removed fibroblasts by using a cell scraper under a microscope. After 3–6 passages, the epithelial cells were used for further experiments.

### Rapamycin treatment and siRNA knockdown

Pterygium epithelial cells generated in 3–6 passages were used for the following experiments. Cells were treated with rapamycin (100 nM), bafilomycin A1 (50 nM) or both for 48 h following protein extraction. *TSC1, Bcl-2* and *p73* siRNA oligonucleotides (*Tsc1* siRNA, sense 5′-CCA AAU CUC AGC CCG CUU UTT--3′ and antisense 5′-AAA GCG GGC UGA GAU UUG GTT-3′; *Bcl-2* siRNA, sense 5′-GGA TGC CTT TGT GGA ACT GTA TT-3′ and antisense 5′-TAC AGT TCC ACA AAG GCA TCC-3′; and *p73* siRNA, sense 5′-CAG GCU CUG AAU GAA AGU ATT-3′ and antisense 5′-GCC UUU GGU UGA CUC CUA UTT-3′) (GenePharma, China) were transfected using Lipofectamine 2000, according to the manufacturer’s instructions (Invitrogen).

### Statistical analysis

The results are presented as the mean ± standard error (SEM). The data in each group were analysed using unpaired, two-tailed Student’s t-tests. The relationships between Bcl-2 and LC3 expression and between p-S6 and FGFR3 expression were analysed using Pearson’s correlation analysis. A p value of <0.05 was considered statistically significant. All statistical analyses were performed with SPSS software, version 13.0.
